# Supplementary material for: Collapse on the line – how synthetic dimensions influence nonlinear effects
Source: Sci Rep. 2019 Jul 2;9:9518. doi: 10.1038/s41598-019-46060-8 (PMC6606584; doi:10.1038/s41598-019-46060-8)
Supplement: Supplementary file 1 — Supplementary Material: Collapse on the line XX how synthetic dimensions influence nonlinear effects [file 41598_2019_46060_MOESM1_ESM.docx]

Collapse on the line – how synthetic dimensions influence nonlinear effects

**André L. M. Muniz^1^, Martin Wimmer^1^, Arstan Bisianov^1^, Roberto Morandotti^2,3,4^ and Ulf Peschel^1^**

*1. Abbe Center of Photonics, Friedrich Schiller University Jena, Max-Wien-Platz 1, 07743 Jena, Germany*

*2.*  *INRS EMT, 1650 Blvd Lionel Boulet, Varennes, PQ J3X 1S2, Canada*

*3. ITMO University, St. Petersburg, Russia*

*4. Institute of Fundamental and Frontier Sciences, University of Electronic Science and Technology of China, Chengdu 610054, China*

Content

[Supplementary Note 1: Experimental setup 2](#_Toc529974720)

[Supplementary Note 2: 2D Time multiplexing 4](#_Toc529974721)

[Supplementary Note 3: Evolution equations 6](#_Toc529974722)

[Supplementary Note 4: Band structure and external phase modulation 7](#_Toc529974723)

[Supplementary Note 5: Broad Gaussian excitations 9](#_Toc529974724)

[Supplementary Note 6: Numerical analysis of the nonlinear solutions 12](#_Toc529974725)

[Supplementary References 14](#_Toc529974726)

## Supplementary Note 1: Experimental setup

The experimental setup consists of two main parts: the signal generation module (see Supplementary Fig. 1) and the time multiplexing part with the coupled fiber loops (see Supplementary Fig. 2). A CW (continuous wave) signal at $\lambda=1550$nm, emitted by a distributed feedback (DFB) laser diode, is cut into a train of rectangular pulses of 22 ns width by using a Mach-Zehnder modulator (MZM). The pulse chain is amplified by two erbium-doped fiber amplifiers (EDFA). Afterward, another MZM removes the space between two consecutive pulses. A tunable optical bandpass filter (BPF) eliminates the additional out-of-band noise-amplified spontaneous emission (ASE) - added by the amplifiers. A variable optical attenuator (VOA) is used for coarse control of the peak power, whereas a fine tuning of the initial peak power is carried out by an acousto-optic modulator (AOM) with variable transmission rate. The AOM is also used for performing an automatized sweep over different power levels and acts as a gate, which only lets a one seed pulse enter the fiber loop system. The tunable band pass filter (BPF) further cleans the input pulses.


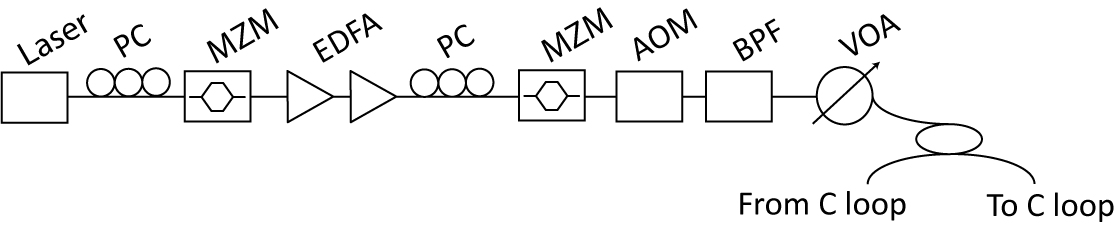


**Supplementary Figure 1.** Signal generation. At the beginning, the CW signal of a laser diode is cut into a train of pulses by a Mac-Zehnder-Modulator (MZM) followed by two erbium-doped fiber amplifiers (EDFA). A second MZM is used for cleaning the background between two successive pulses. An acousto-optic modulator (AOM) acts as a gate to the fiber loops and provides a variable transmission ratio, which is necessary for performing an automatized sweep over different power levels. For a manual control of the peak power, a variable optical attenuator (VOA) is inserted at the end after a tunable bandpass filter (BPF). Polarization controllers are denoted by three red circles. Finally, the pulse is coupled into the loop by a 50/50 coupler.

The generated 22 ns long pulse is injected into the system of coupled fiber loops by a 50/50 optical coupler, as shown in Supplementary Fig 2. The experimental platform is built up of four coupled fiber loops of slightly different lengths, arranged in a pair of inner and outer loops connected by two 50/50 couplers. Within each loop, an EDFA compensates for the overall losses that accumulate every round trip. In order to adjust the amplification factors of the EDFAs and for avoiding any transients, a pilot CW laser operated at a blue-shifted wavelength ($\lambda=1536$ nm) is inserted into each EDFA via a wavelength division multiplexing (WDM) coupler. The pilot signals are removed by tunable BPFs directly after the amplifiers.

Phase modulators (PMs) and amplitude modulators (AOM and MZM) can control the phase and amplitude, respectively, of each pulse in the inner and outer loops. The amplitude modulators are adjusted for a transmission ratio of 0.9 in the passive case, which is exactly balanced by the amplification of the EDFAs, and enable a dynamic gain and loss control for creating Gaussian distribution excitation. The polarization state of light is controlled by two additional photodetectors coupled via polarizing beam splitter (PBS) in the outer loops. Minimizing the signals at these photodetectors ensure a stable polarization state of the pulses for more than 120 roundtrips and guarantees a proper operation of polarization maintaining components, such as PM and MZM. As depicted in Supplementary Fig. 2, AOM are placed at the beginning of each inner loop, but MZMs in the outer ones. The AOMs provide a higher suppression ratio and requires only an RF signal. Although having longer switching times compared with MZMs, they are still fast enough for controlling pulses on time scales of the order of microseconds. Additionally, the performance of the AOMs does not depend on the state of polarization, which simplifies the set-up considerably. Likewise, MZMs in the outer loops provide much faster control of pulses in the nanoseconds range but require DC bias control and polarization stability. It is very important to control the polarization of pulses and in particular that those arriving at a beam splitter after travelling through different loops have the same polarization state and can coherently interfere.

All loops consist of standard single-mode fibers (SSMF) combined with $\approx3 \text{km}$ of dispersion-compensating fibers (DCF, type: OFS-HSDK, nonlinear coefficient $\gamma\approx7 \text{W}^{-1}\text{km}^{-1}$ [S1]) to ensure an overall normal dispersion in the loops to avoid modulational instabilities. In addition, these dispersion compensating fibers boost nonlinear effects such that a nonlinear phase shift of $2\pi$ is achieved for pulses with an input peak power of about $157 \mathrm{mW}$[S2]. Loop pairs are built up in the most symmetric way by arranging amplitude modulators, EDFAs, tunable BPF, photodetectors (PDs), DCFs, SSMFs and PMs in the same way.

**
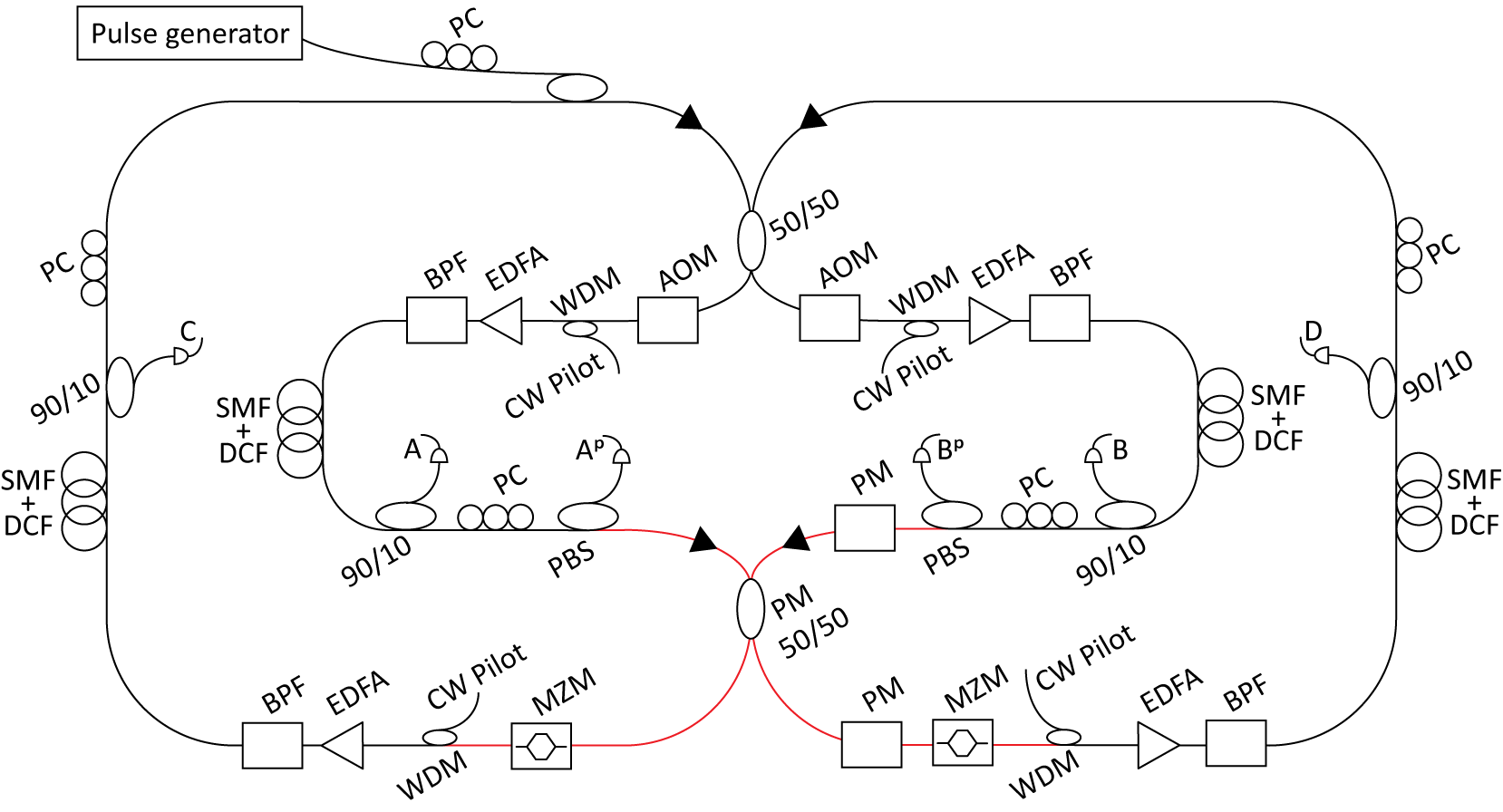
**

**Supplementary Figure 2.** Two pairs of fibers are connected via 50/50 couplers (see upper panel). A pulse is created and injected into the outer left loop. Each fiber path has an erbium-doped fiber amplifier (EDFA) for loss compensation and about 30 km of optical fiber. Acousto-optic modulators (AOM) in the inner and Mach-Zehnder modulators (MZM) in the outer pair allow for amplitude modulation. Additionally, phase modulators (PM) are placed in the inner and outer right loop. Red lines represent polarization maintaining patch cords.

## Supplementary Note 2: 2D Time multiplexing

Here, the time multiplexing principle discussed in [S3, S4] and demonstrated for Light Walks in [S5] is adapted to 2D Light Walks. The fundamental working principle of this technique is based on the idea, that the 2D coordinates $\left( x,y \right)$ on the lattice are encoded together with the temporal evolution on a single temporal dimension. This is achieved by coupling four fiber loops of different length as shown in Supplementary Fig. 2. All fiber patches are approximately $30 \mathrm{km}$ long, in which the two outer (C and D) ones differ by 6 m and the two inner ones (A and B) by $600 m$. During one roundtrip, one inner and one outer loop has to be passed, which results in a total travel length of about $60 \mathrm{km}$ or a round trip time of $\overline{T}=300 \mu\text{s}$. However, due to the different fiber lengths, pulses arrive after one round trip at different time slots, which encodes the travelled distance. After one roundtrip, the pulse that took the shorter inner and outer loop arrives first, whereas the pulse that propagated through the longer inner and outer loop arrives delayed. Thus, the arrival time

$$T_{\text{arrival}}=m\underset{\overline{T}}{\underbrace{\left( \frac{T_{A}+T_{B}}{2}+\frac{T_{C}+T_{D}}{2} \right)}}+\underset{{\Delta T}_{\text{inner}}}{\underbrace{\frac{T_{A}-T_{B}}{2}}}x+\underset{{\Delta T}_{\text{outer}}}{\underbrace{\frac{T_{C}-T_{D}}{2}}}y=m\overline{T}+\Delta T_{\text{inner}}x+\Delta T_{\text{outer}}y$$

is given by a linear combination of the roundtrip time$\overline{T}$and the length differences $\Delta T_{\text{inner}}$ and ${\Delta T}_{\text{outer}}$. Here, $x$,$y$ and $m$ denote integer numbers, where $m$ counts the number of roundtrips and $x$,$y$ are increased or decreased after each roundtrip through the longer or shorter patch of the inner and outer loops. In the example above, the earliest pulse taking the shorter inner and outer loop arrives at $T_{\text{arrival}}=T_{B}+T_{D}$ and thus $x=-1$ and $y=-1$, which is equal to a step to the left bottom on the 2D lattice. The latest pulse arrives at $T_{\text{arrival}}=T_{A}+T_{C}$, which corresponds to $x=1$ and $y=1$ (right top on the lattice). Taking the inner short and outer long loop leads to a delay of $T_{\text{arrival}}=T_{B}+T_{C}$, which equals $x=-1$ and $y=1$ (left top). The last case of a roundtrip through the inner long and outer short loop results in $T_{\text{arrival}}=T_{A}+T_{D}$ ($x=1$ and $y=-1$, or right bottom on the 2D lattice). Therefore, the 2D lattice can be created by time multiplexing as depicted in Supplementary Fig. 3.

The size of the mesh lattice displayed in Supplementary Fig. 3 is limited by the used fiber lengths: If the earliest pulse from roundtrip $m+1$overlaps with the latest pulse from roundtrip$m$, the pulses from two subsequent roundtrips cannot be distinguished any more. Therefore, the maximum number of positions along the horizontal direction is approximately given by $x_{\text{max}}\approx\overline{T}/{\Delta T}_{\text{inner}}$. In the same way, time multiplexing fails, if during one roundtrip two pulses from adjacent medium time slots overlap. This results in a maximum vertical lattice size $y_{\text{max}}\approx{\Delta T}_{\text{inner}}/\Delta T_{\text{outer}}$.

As only pulses arriving at the same time at a 50/50 coupler can interfere, only those pulses interact which have performed the same number of passages through the different loops, but potentially in a different order. For example, pulses propagating first through the shorter inner and outer loop and afterwards through the longer inner and outer long loop arrive at the same time as the pulses that first took the longer inner and outer loop and in the second time step the shorter inner and outer patches. Hence, those pulses finally arrive on the same lattice point, but following different trajectories. Since always the same components are passed, any phase disturbance with a time scale smaller than the measurement time influences all pulses in the same way and therefore, the system acts as a self-aligning interferometer.


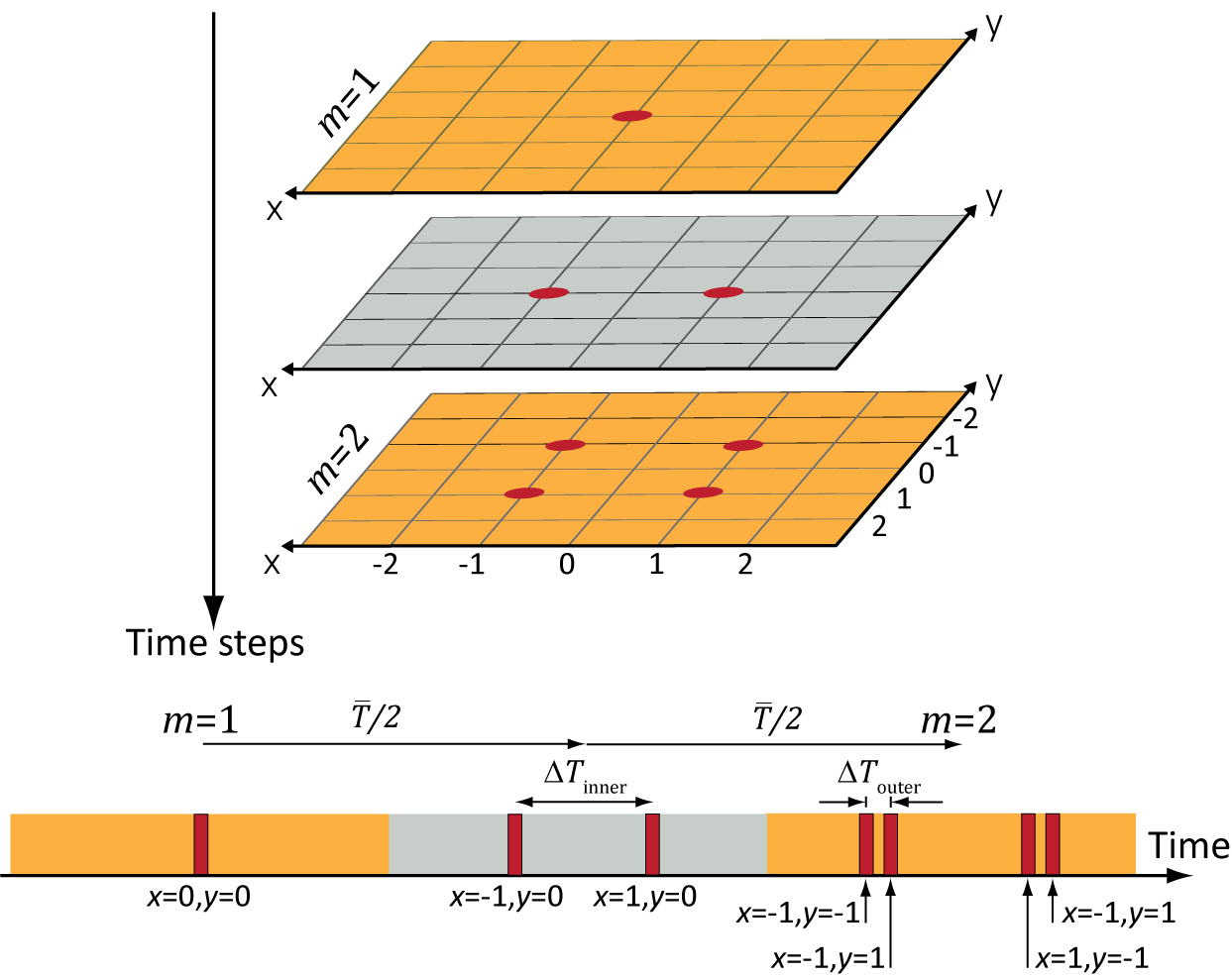


**Supplementary Figure 3.** Mapping of pulses onto a 2D lattices based on their arrival times. The time traces measured at photodiodes (lower panel, pulses correspond to red stripes) are mapped step by step onto a 2D lattice (upper part of the figure)

## Supplementary Note 3: Evolution equations

Each of the used 50/50 coupler is described by a $2\times2$ matrix

$\hat{C}=\frac{1}{\sqrt{2}}\left( \begin{matrix} 1 & i \\ i & 1 \end{matrix} \right),$ (1)

where the reflected port acquires a phase shift of $\pi/2$. Since dispersion is negligible for pulses with a length of 22 ns and overall propagation distances of not more than $6000 \mathrm{km}$, the system is mathematically described by the average pulse amplitudes $a_{x,y}^{m}$, $b_{x,y}^{m}$, $c_{x,y}^{m}$ and $d_{x,y}^{m}$ for loops from A to D during roundtrip $m$ at position $\left( x,y \right)$. After passing the inner shorter (B) and longer loop (A), the horizontal coordinate $x$ is decreased (B) or increased (A) by one. Afterwards, the pulses are coupled according to the beam splitter matrix in Supplementary Eq. (1). Hence, the first part of the round trip represents a step to the left ($b_{x,y}^{m}$) and right ($a_{x,y}^{m}$) on the mesh lattice

$a_{x,y}^{m}=\frac{1}{\sqrt{2}}\left( c_{x-1,y}^{m-1}+id_{x-1,y}^{m-1} \right)e^{i\chi\left| c_{x-1,y}^{m-1}+id_{x-1,y}^{m-1} \right|^{2}}$ and (2)

$b_{x,y}^{m}=\frac{1}{\sqrt{2}}\left( d_{x+1,y}^{m-1}+ic_{x+1,y}^{m-1} \right)e^{i\chi\left| d_{x+1,y}^{m-1}+ic_{x+1,y}^{m-1} \right|^{2}}e^{i\varphi(m)}$, (3)

whereas pulses travelling afterwards through loops C and D move downwards ($y$ is decreased by one) or upwards ($y$ is increased by one). This results in a second set of evolution equations

$c_{x,y}^{m}=\frac{1}{\sqrt{2}}\left( a_{x,y-1}^{m}+ib_{x,y-1}^{m} \right)e^{i\chi\left| a_{x,y-1}^{m}+ib_{x,y-1}^{m} \right|^{2}}$ and (4)

$d_{x,y}^{m}=\frac{1}{\sqrt{2}}\left( b_{x,y+1}^{m}+ia_{x,y+1}^{m} \right)e^{i\chi\left| b_{x,y+1}^{m}+ia_{x,y+1}^{m} \right|^{2}}e^{i\varphi(m)}$. (5)

for the fields $c_{x,y}^{m}$ and $d_{x,y}^{m}$. After passing loops C and D, the pulses have accomplished one round trip and therefore m is increased by 1*.* Note that a pulse has to perform two round trips to return to its origin. Here $\varphi_{0}$ a phase shift introduced by phase modulators in loops B and D and $\chi$ denotes an effective nonlinearity $\chi=\gamma L^{eff}$, where $\gamma$ and $L^{eff}$ are fiber nonlinear coefficient and effective fiber length, respectively. Experimentally, we employ 30 km of standard single-mode fiber (SSMF; $\gamma\approx1 \text{W}^{-1}\text{km}^{-1}$) combined with $3 \text{km}$ of dispersion-compensating fiber ($\gamma\approx7 \text{W}^{-1}\text{km}^{-1}$ [S1]) in order to enhance nonlinear phase shift of those pulses propagating through the fiber loops. In this model, the pulses first acquire a nonlinear phase shift proportional to their optical power during propagation through the fiber spools. Afterwards, a linear phase shift $\exp\left[ i\varphi\left( m \right) \right]$ is applied in loop B and D by phase modulators and finally inner and outer loops are coupled again with a ratio of 50/50.

## Supplementary Note 4: Band structure and external phase modulation

In order to calculate the band structure of the unmodulated lattice ($\varphi=0$) in the linear regime$\left( \chi=0 \right)$, we first apply a simplification on Supplementary Eqs. (2)-(5) in order to reduce into two iteration equations. As $a_{x,y}^{m}$ and $b_{x,y}^{m}$ are uniquely determined by $c_{x,y}^{m-1}$ and $d_{x,y}^{m-1}$ and those depend on $a_{x,y}^{m-1}$ and $b_{x,y}^{m-1}$ only, it is possible to replace $c_{x,y}^{m-1}$ and $d_{x,y}^{m-1}$ as

$a_{x,y}^{m+1}=\frac{1}{2}\left\{ a_{x-1,y-1}^{m}+ib_{x-1,y-1}^{m}+\left( ib_{x-1,y+1}^{m}-a_{x-1,y+1}^{m} \right)\exp\left[ i\left( -1 \right)^{m-1}\varphi_{0} \right] \right\} \mathrm{and}$ (6)

$b_{x,y}^{m+1}=\frac{1}{2}\left\{ \left( b_{x+1,y+1}^{m}+ia_{x+1,y+1}^{m} \right)\exp\left[ i\left( -1 \right)^{m-1}\varphi_{0} \right]+ia_{x+1,y-1}^{m}-b_{x+1,y-1}^{m} \right\}.$ (7)

Furthermore, we use, as in the 1D counterpart, a Floquet-Bloch ansatz [S6]

$\left( \begin{aligned} a_{x,y}^{m} \\ b_{x,y}^{m} \end{aligned} \right)=\left( \begin{aligned} A \\ B \end{aligned} \right)e^{i(k_{\text{x}}x+k_{\text{y}}y-\theta m)/2}$, (8)

where $k_{\text{x}}$ and $k_{\text{y}}$stand for Bloch momenta in $x$ and $y$ directions, and $\theta$ is the propagation constant. In Supplementary Eq. (8), $\left( A,B \right)^{\text{t}}$ is the eigenvector consisting of two components, which describe the amplitude and phase relation between loops A and B. The structure of the ansatz accounts for the double periodicity of our lattice, i.e. a unit cell contains two points in each direction and two round trips are required to reproduce the lattice. Inserting the ansatz into a double step of the evolution equations Supplementary Eqs. (2)-(5) yields the dispersion relation:

$\cos\left( \theta\right)=\pm\frac{1}{2}[-1-\cos\left( k_{x} \right)-\cos\left( k_{y} \right)+\cos\left( k_{x} \right)\cos\left( k_{y} \right)]$, (9)

which consists of two bands connected by Dirac cones as depicted in Supplementary Fig 4a. As solitons are localized states with exponentially decaying tails, their propagation constant lies within the band gap of a system. Consequently, the 2D mesh lattice has to be modulated in order to open up a band gap for observing self-localization. For this reason, a phase modulation

$\varphi(m)=\left\{ \begin{aligned} -\varphi_{0}, &\text{odd} m \\ {+\varphi}_{0}, &\text{even} m \end{aligned} \right.$ (10)

with alternating sign is applied to loops B and D. Inserting the phase modulation in Supplementary Eq. (10) into the evolution equations Supplementary Eqs. (2)-(5) and using the Floquet-Bloch ansatz in Supplementary Eq. (8), results in the dispersion relation

$\cos\left( \theta\right)=\pm\frac{1}{2}[-\frac{1}{2}-\frac{1}{2}\cos\left( 2\varphi\right)-cos(\varphi)\cos\left( k_{x} \right)-cos(\varphi)\cos\left( k_{y} \right)+\cos\left( k_{x} \right)\cos\left( k_{y} \right)]$. (11)

For the passive case ($\varphi_{0}=0$), Supplementary Eq. (11) simplifies to Supplementary Eq. (9), however for any non-vanishing $\varphi_{0}$ the degeneracy at the Dirac cone at the edge of the Brillouin zone is lifted and a gap opens up as shown in Supplementary Fig. 4b-f. A new Dirac cone is formed in the center of the Brillouin zone for$\varphi_{0}=\pm\pi$ (Supplementary Fig. 4g). Furthermore, due to the Floquet-Bloch nature of the system, the passive band structure repeats itself with respect to $\theta$ for multiples of$2\pi$.

| 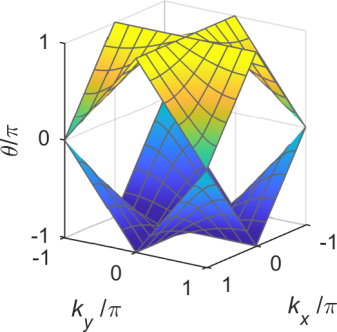 | 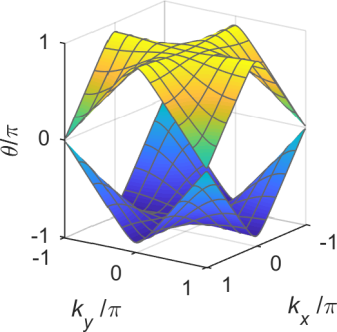 | | 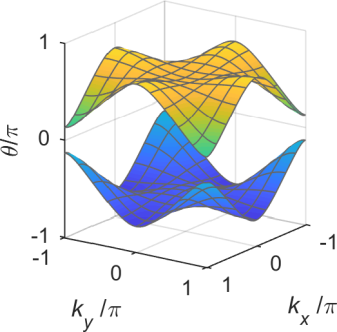 | | 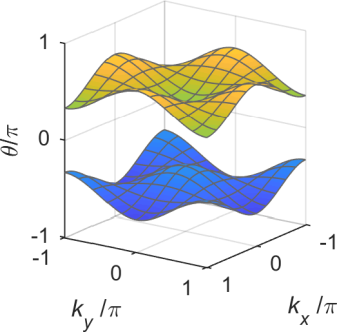 |  |  |
| --- | --- | --- | --- | --- | --- | --- | --- |
| (a) $\varphi= 0$ | (b) $\varphi= \pi/6$ | | (c) $\varphi= \pi/3$ | | (d) $\varphi= \pi/2$ |  |  |
| 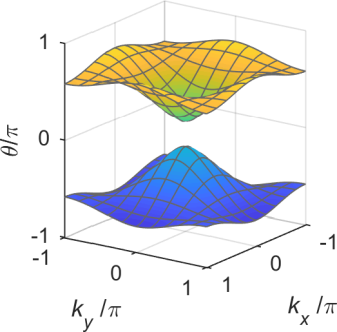 | | | 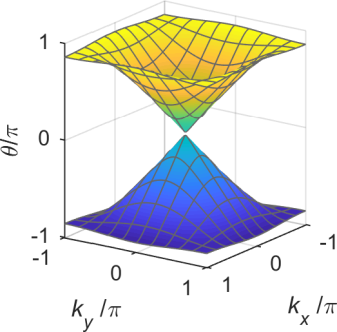 | | 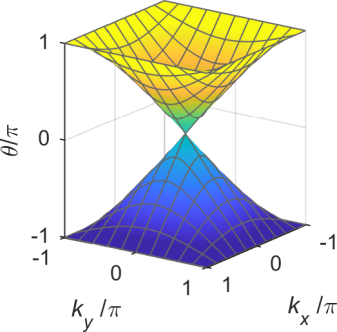 | | |
| (e) $\varphi= 2\pi/3$ | | | (f) $\varphi= 5\pi/6$ | | (g) $\varphi= \pi$ | | |
| **Supplementary Figure 4.** 2D band structure as a function of the phase modulation. | | | | | | | |
|  | | | | | | | |

## Supplementary Note 5: Broad Gaussian excitations

In contrast to a single lattice site excitation, which populates the complete band structure including the upper and lower band, a specific point of the Brillouin zone is excited by a spatially broad wave packet with a narrow momentum spread. Here, we use a chain of rectangular pulses which altogether form a Gaussian envelope with a $1/e$ width of 6 positions. However, inserting an externally generated wave packet requires an active interferometric stabilization. In order to circumvent this technical challenge, a protocol is used for creating pulse chains with a Gaussian envelope, which is explained in detail in [S6] and [S7] for the 1D system. The working principle of this experimental protocol is based on the fact, that blocking inner and outer loops alternately every time step results in diffusive equations, as explained in the following for the linear system$(\chi=0$) without external phase modulation ($\varphi_{0}=0$).

At time step$m=1$, a single pulse is inserted into the system. For $m=0$ until $m=M$ time steps, loop A and B are blocked in an alternating way resulting in the following evolution equations:

$$a_{x,y}^{m+1}=\frac{1}{2}\left( \underset{=0, \text{for} \text{even}(m)}{\underbrace{a_{x+1,y+1}^{m}}}+\underset{=0, \text{for} \text{odd}(m)}{i\underbrace{b_{x+1,y+1}^{m}}}+\underset{=0, \text{for} \text{odd}(m)}{i\underbrace{b_{x+1,y-1}^{m}}}-\underset{=0, \text{for} \text{even}(m)}{\underbrace{a_{x+1,y-1}^{m}}} \right),$$

$$b_{x,y}^{m+1}=\frac{1}{2}\left( \underset{=0, \text{for} \text{odd}(m)}{\underbrace{b_{x-1,y-1}^{m}}}+\underset{=0, \text{for} \text{even}(m)}{i\underbrace{a_{x-1,y-1}^{m}}}+\underset{=0, \text{for} \text{even}(m)}{i\underbrace{a_{x-1,y+1}^{m}}}-\underset{=0, \text{for} \text{odd}(m)}{\underbrace{b_{x-1,y+1}^{m}}} \right).$$

For a double step starting at$m=0$, this results in a diffusive equation

$$a_{x,y}^{m+2}=\frac{i}{4}\left( b_{x+2,y+2}^{m}-b_{x+2,y-2}^{m} \right),$$

$$b_{x,y}^{m+2}=-\frac{1}{4}\left( b_{x,y-2}^{m}+2b_{x,y}^{m}+b_{x,y+2}^{m} \right).$$

along the $y$-direction (see Supplementary Fig. 5a-c). After the first $M$ time steps, the same scheme is applied to loops C and D while A and B are not modulated anymore:

$$c_{x,y}^{m+1}=\frac{1}{2}\left( \underset{=0, \text{for} \text{even}(m)}{\underbrace{c_{x+1,y+1}^{m}}}+\underset{=0, \text{for} \text{odd}(m)}{\underbrace{id_{x+1,y+1}^{m}}}+\underset{=0, \text{for} \text{odd}(m)}{\underbrace{id_{x-1,y+1}^{m}}}-\underset{=0, \text{for} \text{even}(m)}{\underbrace{c_{x-1,y+1}^{m}}} \right),$$

$$d_{x,y}^{m+1}=\frac{1}{2}\left( \underset{=0, \text{for} \text{odd}(m)}{\underbrace{d_{x-1,y-1}^{m}}}+\underset{=0, \text{for} \text{even}(m)}{\underbrace{ic_{x-1,y-1}^{m}}}+\underset{=0, \text{for} \text{even}(m)}{i\underbrace{c_{x+1,y-1}^{m}}}-\underset{=0, \text{for} \text{odd}(m)}{\underbrace{d_{x+1,y-1}^{m}}} \right).$$

For a double step starting from an even $m=M$ this results in diffusion equations

$$c_{x,y}^{m+2}=\frac{i}{4}\left( d_{x+2,y+2}^{m}-d_{x-2,y+2}^{m} \right)$$

$$d_{x,y}^{m+2}=-\frac{1}{4}\left( 2d_{x,y}^{m}+d_{x-2,y}^{m}+d_{x+2,y}^{m} \right)$$

along the horizontal direction (see Supplementary Fig. 5d-f). In this way, after $M$ time steps along the vertical and another $M$ time steps along the horizontal direction, a broad Gaussian distribution

$$a_{x,y}^{m}=\frac{1}{\sqrt{2M}}e^{-\frac{x^{2}+y^{2}}{4M}}$$

is created, while preserving the intrinsic stabilization of the system.

In time step $2M-1$, one of the loops C and D is blocked for the last time and consequently, when passing next time the 50/50 at the beginning of time step $m=2M$, the Gaussian distribution is split with an equal intensity into two parts propagating through loops A and B. A phase shift of $\pi/2$ introduced by the fiber coupler (see Supplementary Eq. (1)) is compensated by the phase modulator in loop D at time step$2M$. Additionally, in order to excite an eigenstate of the system, an amplitude and phase relation has to be adjusted between loops A and B at the time step $2M$ by the modulators according to the eigenstate in Supplementary Eq. (8) and depicted in Supplementary Table 1. For instance, considering$\varphi_{0}=\pi/2$, the eigenstate for exciting the center (*k_x_* = *k_y_* = 0) of the focusing band should have an amplitude ($\left| A \right|/\left| B \right|$) and phase ($\arg A/B$) relation of $1.93185$and $- \pi/4$ respectively.


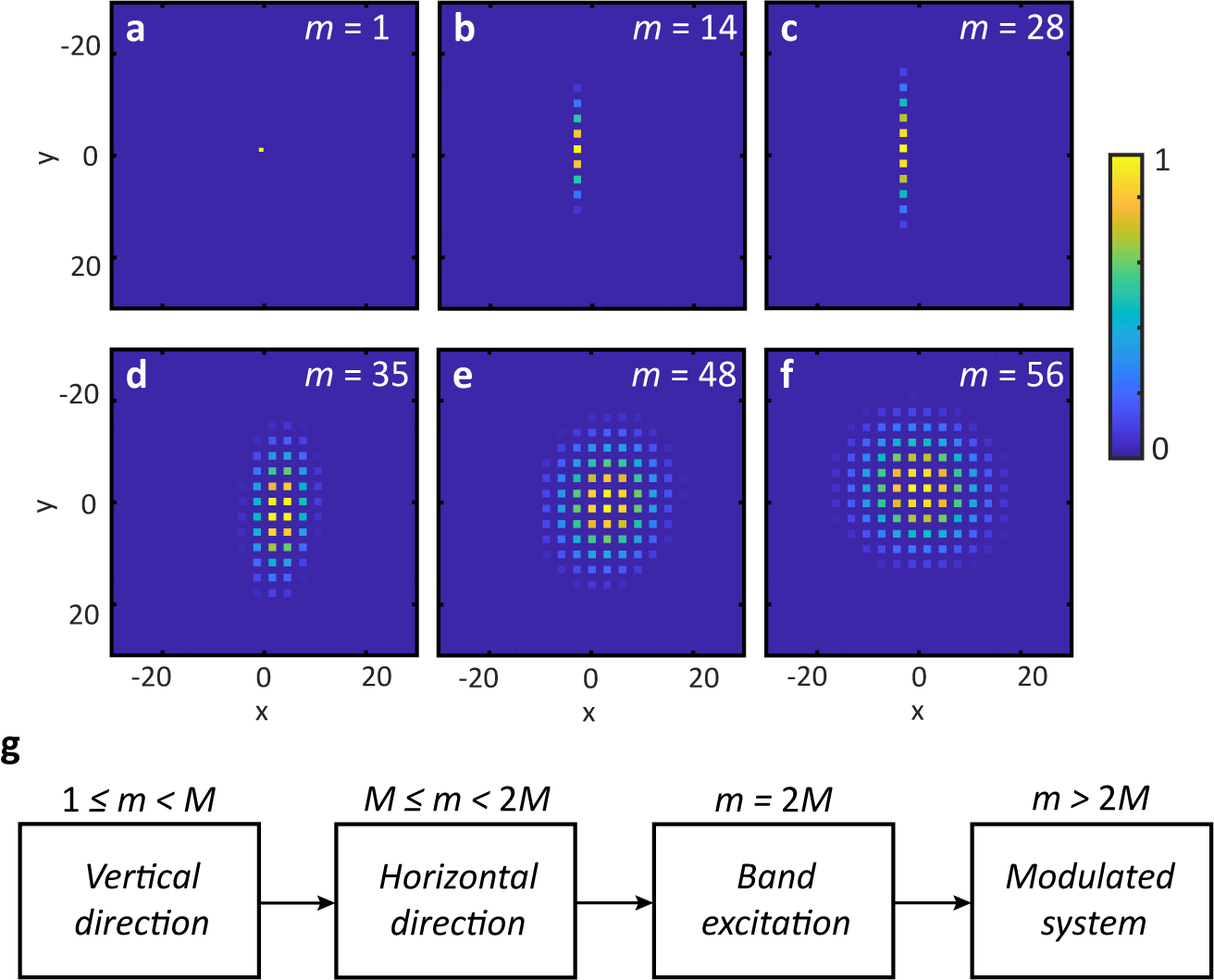


**Supplementary Figure 5.** Protocol used for creating pulse chains with a Gaussian envelope. **a-c**, Discrete points with a Gaussian envelope expands in $y$-direction. **d-f**, A Gaussian envelope expands in $x$-direction. **g**, Flowchart of the preparation and measurement set for Gaussian beam analysis.

**Supplementary Table 1.** Amplitude and phase difference between loop A and B at the time step 2*M* in order to excite the center of the Brillouin Zone (*k_x_* = *k_y_* = 0) for different modulated system configurations.

| $\varphi_{0}$ | $\left\vert A \right\vert/\left\vert B \right\vert at m=2M$ | $\arg(A/B)at m=2M$ |
| --- | --- | --- |
| *φ* = π/6 | 2.35625 | $- 5\pi/12$ |
| *φ* = π/3 | 2.1889 | $- \pi/3$ |
| *φ* = π/2 | 1.93185 | $- \pi/4$ |
| *φ* = 2π/3 | 1.61803 | $- \pi/6$ |
| *φ* = 5π/6 | 1.29177 | $- \pi/12$ |

## Supplementary Note 6: Numerical analysis of the nonlinear solutions

Here, we numerically analyze the localized nonlinear solutions of equations (2)-(5), arising from the focusing ($\theta>0$) band at $\chi>0$ and$\varphi_{0}=\pi/2$. In accordance with experimental conditions the size of the computational domain was 80 positions in both $x$ and $y$ directions and periodic boundary conditions were used for termination. To find the solutions in an iterative way, we used an in-built MatLab iterative algorithm (“trust-region-dogleg”) based on “trust regions” with the Jacobian matrix derived numerically from the objective function. Throughout the calculation, $\chi$ was fixed to one, while the field intensity was kept unconstrained.

**Unstable Townes-like soliton**

First, we concentrate on the unstable Townes-like soliton that generally collapses or broadens if its energy fluctuates towards a higher or lower value, respectively. Width$w$, amplitude $A$ (here we set 𝜒 to 1) and propagation constant $\theta$ of the soliton can vary. If $w\gg1$, the soliton enters the continuous limit, where it can be well described by the 2D Nonlinear Schrödinger equations (NLS) and thus becomes the genuine Townes soliton. Since the latter has an intensity profile very similar to the Gaussian function$G_{w}(x,y)=exp\left[ -\left( x^{2}+y^{2} \right)/{w^{2}} \right]$, we use as a trial

$\left( \begin{matrix} a_{x,y}^{m=0} \\ b_{x,y}^{m=0} \end{matrix} \right)=A_{w}G_{w}(x,y)\frac{1}{\sqrt{1+\left| \sigma\right|^{2}}}\left( \begin{matrix} \sigma\\ 1 \end{matrix} \right)$,

where we approximate the field distribution in the loops by the eigenvector of the lower band at the center of the 1st Brillouin zone and thus use as given by Supplementary Table (1). The corresponding linear eigenvalue is $\theta_{c}=\pi/3$ according to the notation introduced in Supplementary Eq. (8). The trial values of $A_{w}$ and $w$ are first guessed for the case of a very weak nonlinearly induced change of the propagation constant $\theta_{w}=\theta_{c}+\delta$. If we assume the soliton to have the double periodicity of the lattice, the following optimization task has to be solved:

$$\left\{ \begin{matrix} a_{x,y}^{m=2}-a_{x,y}^{m=0}e^{i\left( \theta_{c}+\delta\right)}\to0 \\ b_{x,y}^{m=2}-b_{x,y}^{m=0}e^{i\left( \theta_{c}+\delta\right)}\to0 \end{matrix} \right. ,$$

where the increment $\delta$ should be much smaller than $\theta_{c}$ and $(a_{x,y}^{m=2},b_{x,y}^{m=2})$ is the field calculated after one full period. After the optimized soliton is obtained, it is further used as a trial for searching a soliton with a slightly higher propagation constant. Next, the obtained solution is again used as a trial for a larger propagation constant, and so on until the edge of the upper band is encountered. Thus, we continuously trace the family of solutions inside the band gap with the width $\Delta\theta=2\pi/3$. The outcome is provided in Supplementary Figure 6.

In general, we found several nonlinear solutions, both stationary and oscillating ones. The stationary solutions return to their initial shape after the shortest recovery period of the lattice, i.e. after two time steps. In contrast, the oscillating ones recover after 4 or even 6 time steps. In the experiments, we always observe the Gaussian excitation to evolve around stationary states and therefore, we restrict to the discussion of these solitary structures. We found two stationary structures: a Townes-like soliton, which tends to collapse and an extremely localized solution which usually marks the final state of the evolution.

In the limit$\theta\to\theta_{c}$, the Townes-like soliton spreads to infinite width and thus converges to its counterpart known from continuous systems. However, as we investigate a limited square domain ($80\times80$) the energy curve even shows a little increase below$\theta\approx0.31\pi$. Unlike the continuous case where the total energy of a Townes soliton is constant and independent of its propagation constant, discreteness causes the energy to increase with growing $\theta$ while the width of the solution and thus the energy portion in the central spike remains almost constant (see Supplementary Fig. 6).

Although stationary, the Townes soliton was found to degrade during propagation as expected. Still its lifetime is the highest close the edge of the upper band $(\theta\lesssim0.3\pi)$ and its intrinsic instability seems to be even boosted by discreteness. In the lower half of the gap any perturbation of the soliton profile results in an almost immediate blow up of the field causing a transformation into a less energetic and more stable highly localized stationary solution. This evolution we observe both in numerical simulations and in experiment.

As a collapse of the field distribution is finally terminated by discreteness above a certain power threshold, we always end up with a highly localized stationary solution. Its power as determined numerically is almost completely concentrated in a central spike. Its total energy grows slowly with increasing propagation constant except a small range of propagation constants close to the lower band edge. Here, the solution appears to be unstable where for all other cases the highly localized states seem to mark the stable end point of the evolution.

**
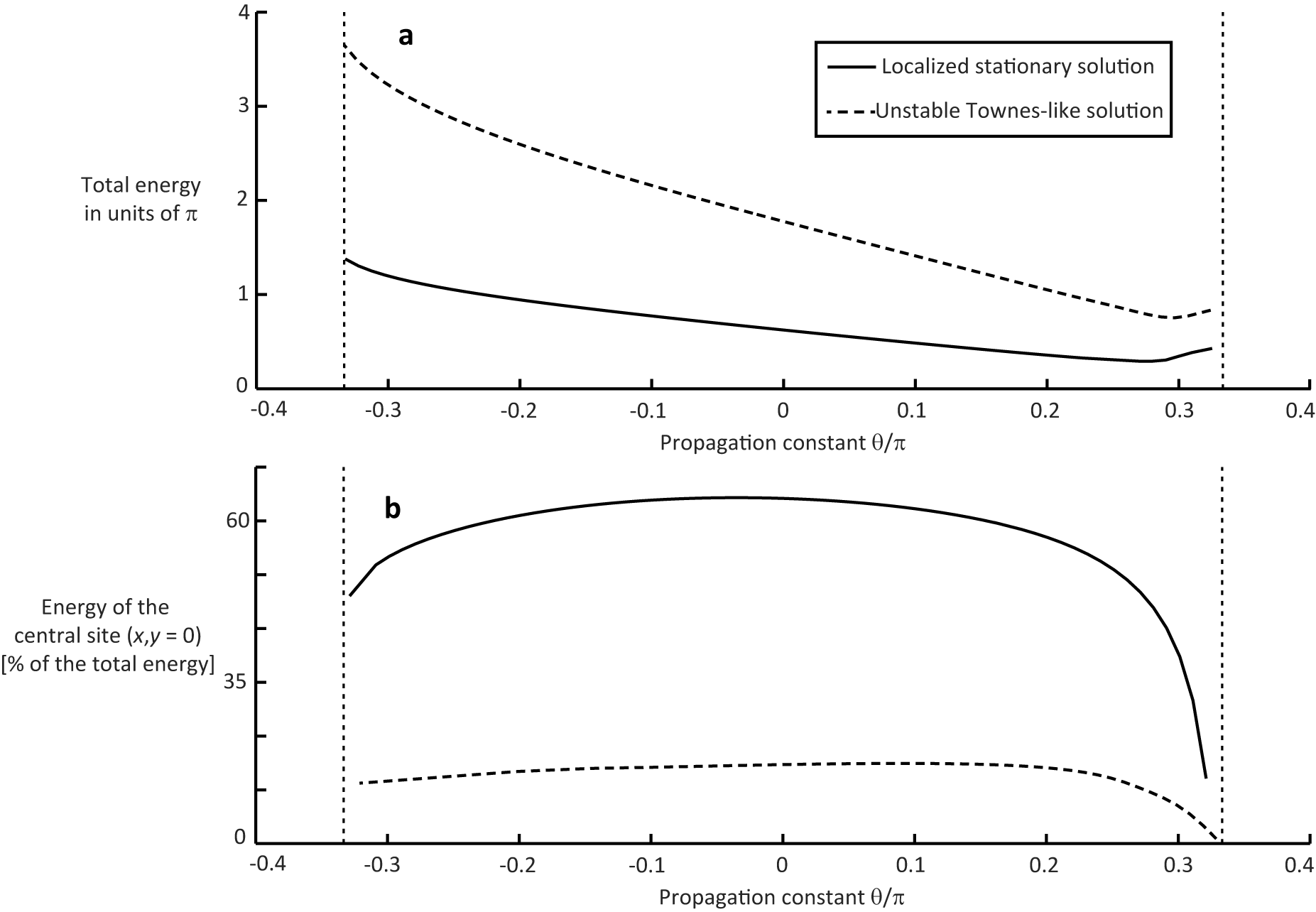
**

**Supplementary Figure 6.** Numerically determined properties of the two types of solitons, which were also observed in the experiment. In all investigations, the nonlinear coefficients $\chi$ was set to unity.

## Supplementary References

##### Gruner-Nielsen, L. *et al*. Dispersion-compensating fibers. *J. Lightw. Technol.* **23**, 3566–3579 (2005).

##### Agrawal, G. P. Nonlinear Fiber Optics. (Academic Press, 2007).

##### Schreiber, A. *et al*. Photons Walking the Line: A Quantum Walk with Adjustable Coin Operations. *Phys. Rev. Lett.* **104**, 50502 (2010).

##### Schreiber, A. *et al*. A 2D Quantum Walk Simulation of Two-Particle Dynamics. *Science* (80-. ). **336,** 55–58 (2012).

##### Regensburger, A. *et al*. Photon Propagation in a Discrete Fiber Network: An Interplay of Coherence and Losses. *Phys. Rev. Lett.* **107,** 233902 (2011).

##### Miri, M.-A., Regensburger, A., Peschel, U. & Christodoulides, D. Optical mesh lattices with PT symmetry. *Phys. Rev. A* **86**, (2012).

##### Wimmer, M. *et al*. Optical diametric drive acceleration through action–reaction symmetry breaking. *Nat. Phys.* **9**, 780–784 (2013).
